# Untangling Triple-Negative Breast Cancer Molecular Peculiarity and Chemo-Resistance: Trailing Towards Marker-Based Targeted Therapies

**DOI:** 10.7759/cureus.16636

**Published:** 2021-07-26

**Authors:** Bushra Kanwal

**Affiliations:** 1 Internal Medicine, Brookdale University Hospital Medical Center, Brooklyn, USA; 2 Lombardi Comprehensive Cancer Center, Georgetown University Medical Center, Washington, D.C., USA

**Keywords:** triple-negative breast cancer, estrogen receptor, progesterone receptor, biomarker, brca1, immune checkpoint inhibitors, human epidermal growth factor receptor-2, parp inhibitors, breast cancer, rna-expression profiling

## Abstract

Triple-negative breast cancer (TNBC), characterized by the absence of estrogen receptor, progesterone receptor, or human epidermal growth factor receptor-2, affects nearly 15% of women with breast cancer. To date, the mainstay of treatment remains chemotherapy, with all the associated consequences, such as the significant toxicity and the suboptimal effect on the five-year survival rates. RNA-expression profiling showed that TNBC is biologically a heterogeneous malignancy. Therefore, predictive biomarkers matched with the diverse subtypes of TNBC could classify patients that would most benefit from a certain targeted treatment. Three biomarker-driven therapies are currently available: poly-adenosine diphosphate (ADP) ribose polymerase inhibitors for patients with germline BReast CAncer gene (BRCA) mutations, atezolizumab combined with nab-paclitaxel for patients expressing programmed death-ligand 1 (PD-L1) on tumor-infiltrating immune cells, and sacituzumab govitecan, an antibody-drug conjugate targeting human trophoblast cell-surface antigen 2 (TROP-2). Identifying predictive biomarkers is crucial for the optimum generation and implementation of targeted agents for TNBC, while further relevant treatments are in the pipeline given the promising results in clinical trials. Finally, newly developed immunotherapies and other targeted agents should also be investigated in earlier stages of the disease, especially in the neoadjuvant setting, broadening the therapeutic application of such regimens.

## Introduction and background

Breast cancer is the most prevalent malignancy in females worldwide, while metastatic breast cancers are the second leading cause of tumor-related deaths in American women. Only in the United States, more than 3.5 million women have been diagnosed with breast cancer [1]. In 2021, it is estimated that approximately 280,000 new cases of invasive breast cancer will be diagnosed in the United States, and about 43,600 deaths will occur [2].

Almost one out of five cases of breast cancer are identified as triple-negative breast cancer (TNBC). This type is characterized by the absence of estrogen and progesterone receptors and the lack of human epidermal growth factor receptor-2 amplification. Unlike hormone receptor-positive or human epidermal growth factor receptor (HER) 2 positive breast cancer, TNBC affects mainly young (age <50 years) African-American women, especially those who carry the BReast CAncer gene (BRCA) 1/2 mutations [3]. This type of breast cancer is particularly aggressive with high mortality, frequent relapses, and extensive metastases. Nevertheless, the management of TNBC is challenging due to its resistance to typical therapies for other types of breast cancer, such as hormonal therapy and HER-2 antagonists treatments [4].

Even with substantial toxicity, frequent relapses, and low survival rates, chemotherapy remains the primary therapeutic approach for triple-negative breast cancer (TNBC). Based on the relevant transcriptomic analyses, TNBC is a biologically heterogeneous tumor with diverse molecular and phenotypical features. This analysis insight to customized two-step approach of first identifying the patient's TNBC subtype with its responsible predictive biomarkers and rendering with designed targeted therapy.

The biomarker-guided therapies have recently been approved, including poly-ADP ribose polymerase inhibitors for patients with germline BRCA mutations and atezolizumab in conjunction with nab-paclitaxel for patients expressing programmed death-ligand 1 (PD-L1) on tumor-infiltrating immune cells. Furthermore, the newly developed antibody-drug conjugate sacituzumab govitecan-hziy is recently approved for cases with metastatic TNBC, which have progressed on at least two previous lines of treatment for advanced disease [5,6].

The identification of specific biomarkers which would facilitate customized treatment decision-making in TNBC has not yet been achieved. Therefore, this review aimed to explore the molecular heterogeneity of TNBC, study the mechanisms of chemo-resistance, and present potential biomarkers for the generation of targeted therapies. By this means, the objective of such treatments is to prevent the development of metastatic disease and, eventually, to increase the survival rates.

## Review

Triple-negative breast cancer

Breast cancer is one of the most common tumors among women demonstrating high mortality rates [7]. TNBC is defined as the subtype of breast cancer and accounts for nearly 20% of diagnosed cases of breast cancers, affecting primarily younger women. Techniques like gene expression and DNA sequencing are employed to classify TNBC [8]. It is one of the most aggressive types linked to high recurrence, metastatic rates, and poor overall survival [9]. 

Molecular peculiarity - molecular classification upon genomic profiling

Typical subclassification of invasive ductal breast cancers is obtained by immunostaining tumor tissues for estrogen receptor (ER), progesterone receptor (PR), HER-1, and HER-2, and various cytokeratins. An immunohistochemical staining proxy based on biomarkers categorizes breast cancer into five significant subtypes: (1) luminal A (ER-positive and/or PR positive, HER-2 negative); (2) luminal B (ER-positive and/or PR positive, HER-2 positive); (3) HER-2 overexpressing (ER-negative, PR negative, HER-2 positive); (4) basal-like (ER-negative, PR negative, HER-2 negative, cytokeratin 5/6 positive and/or epidermal growth factor receptor {EGFR} positive); and (5) normal breast-like tumors (unclassified) were negative for all five biomarkers. Basal-like breast cancer is differentiated from other TNBC (ER, PR, and HER-2 negative) by expressing cytokeratin 5/6 and/or EGFR. Triple-negative breast cancer is diagnosed through immunohistochemistry (IHC). Nevertheless, the diagnosis is challenging due to the molecular overlap between the triple-negative phenotype and the basal-like subtype of breast cancer (Table 1). Because basal-like breast cancers are usually triple-negative, there is a mistaken belief that these two terms are synonymous. Yet, 70-80% of TNBC are basal-like, whereas approximately 70% of basal-like tumors are triple-negative [8,10,11]. 

**Table 1:** Characteristics of molecular subtypes of breast cancer Pos/Neg: positive/negative; HER-2: human epidermal growth factor receptor 2; ER: estrogen receptor; PR: progesterone receptor

Molecular Subtypes	ER	PR	HER-2	Cytokeratin 5 and 6	Prevalence
Luminal A	Positive	Positive	Negative	Negative	~40%
Luminal B	Positive	Positive	Pos/Neg	Negative	~20%
HER-2 enriched	Negative	Negative	Positive	Negative	~10-15%
Basal-like	Negative	Negative	Negative	Positive	~15-20%
Unclassified	Negative	Negative	Negative	Negative	~2-8%

Treatment challenges - a deviation from novel breast cancer therapy

Like almost any epithelial cancer, breast cancer correlates with more favorable therapeutic and survival outcomes when detected early. Nevertheless, even if breast cancer is diagnosed early, the outcomes may vary across the different molecular subtypes. Usually, the ER-positive breast cancer subtypes, such as luminal A and luminal B, demonstrate a good prognosis with optimal five-year survival rates ranging between 80% and 85%, while the ER-negative subtypes (HER-2-positive and basal-like) are more challenging in their treatment and are linked to poor prognosis with a 50-60% five-year survival rate (Table 1). This variance of outcomes could be partially attributed to the different responses of ER-positive subtypes to anti-estrogenic regimens. Targeted treatment of HER-2 overexpressing breast cancers, like luminal B or HER-2-positive/ER-negative carcinomas, with trastuzumab (Herceptin), either concurrent or sequential with adjuvant chemotherapy, has enhanced survival for patients with these breast cancer subtypes [12,13].

TNBC is characterized by an autonomous growth independently of the expression of hormone receptors. Given, thus, the absence of those receptors, medications like tamoxifen (targeting ER) and trastuzumab (targeting HER-2) do not affect patients with this type of breast cancer. Their variable response to chemotherapy and the high incidence of recurrences justifies the fact that TNBCs demonstrate poor clinical outcomes, overall poor prognosis, and shorter long-term survival. Therapeutic resistance coupled with the lack of proper molecular targets and poor prognosis makes detecting biomarkers and relevant therapeutic targets in triple-negative breast cancers of utmost importance [14,15].

TNBC chemotherapy

Chemotherapy is the first-line treatment for TNBC in the neoadjuvant, adjuvant, or metastatic setting. Taxane and anthracycline-based therapies constitute the primary therapeutic option in TNBC management, whereas platinum-based chemotherapy may also play a role in neoadjuvant and metastatic cases. Although TNBC may be aggressive, up to 40% of patients obtain a complete clinical response (CR) with the concurrent histopathological response at the time of surgery after neoadjuvant chemotherapy, exhibiting higher survival rates. Nevertheless, patients with residual malignant tissues after neoadjuvant chemotherapy have a six-fold likelihood to experience a relapse and a twelve-fold metastatic mortality rate [16].

TNBC chemoresistance 

Although neoadjuvant chemotherapy is beneficial for patients with TNBC, almost half of them may develop resistance and, consequently, poor overall survival. The genomic and molecular origin of this resistance of TNBC remains unclear, partly because there is still a lack of efficient techniques that can determine the intratumor heterogeneity and identify genomic information in unique subpopulations [17,18].

Previous studies on the chemo-resistance of TNBC have concentrated chiefly on in situ hybridization techniques and bulk genomic profiling procedures [19,20]. Using targeted cytogenetic biomarkers, research has shown that genetic diversity did not alter in response to neoadjuvant chemotherapy; on the contrary, it appeared selected for mesenchymal phenotypes [19]. Studies were conducted utilizing next-generation sequencing techniques to profile post-chemotherapy resistant TNBC samples to recognize several clinically meaningful mutations [20]. Single-cell DNA and RNA sequencing procedures can function as valuable tools for resolving intratumor heterogeneity, reforming evolutionary lineages, and identifying uncommon subpopulations [21,22].

Biomarker-based targeted therapies

Chemoresistant TNBC demonstrates genetic diversity, high heterogeneity, and rapid progression, making customized treatment for incomplete responders and relapsed cases a daunting task. Several FDA-approved regimens targeting programmed cell death protein-1 (pembrolizumab) and programmed death ligand-1 (atezolizumab), poly adenosine diphosphate (ADP)-ribose polymerase (olaparib and talazoparib), and/or antibody-drug conjugates (sacituzumab govitecan) have provided promising clinical results for specific patients with TNBC. These inhibitors targeting key genetic mutations and certain molecular signaling pathways that control carcinoma growth have been employed as single agents and/or in conjunction with typical chemotherapy drugs [23]. Next, we present the biomarkers that were involved in the development of novel approved treatments in TNBC. 

BRCA1 and BRCA2 genes - PARP inhibitors

Compared to other subtypes of breast cancer, patients with TNBC show a significant prevalence of germline BRCA mutations (gBRCAm), reaching up to 31% [24]. BRCA1 and BRCA2 are autosomal dominant and tumor suppressor genes that preserve genome integrity. Both genes are involved in the homologous recombination repair (HRR) of DNA. In cells with BRCA1/2 deficiency, DNA double-strand breaks (DSBs) repair depend on poly adenosine diphosphate-ribose polymerase (PARP)1 protein [25]. PARP is an abundant, constitutively expressed nuclear enzyme that promotes DNA repair, cellular proliferation, and signaling to other critical cell-cycle proteins and oncogenes. Wherever there is DNA damage, PARP stimulates intracellular signaling pathways that regulate DNA repair and, therefore, cell survival [26]. As a consequence, inhibition of PARP1 coupled with BRCA1 and BRCA2 deficiency may result in serious, highly selective toxicity in these tumor cells [27].

Lately, PARP inhibitors have been comprehensively investigated as a targeted treatment for geminal BRCA mutations (gBRCAm) in ovarian and breast cancer. PARP inhibitors, such as olaparib, under the brand name Lynparza, and talazoparib, under the brand name Talzenna, have been approved for the management of advanced HER-2-negative breast cancer in individuals with a BRCA1 or BRCA2 mutation [28].

In January 2018, based on the beneficial outcomes from the OlympiAD trial, the FDA approved olaparib for the treatment of cases with deleterious or potentially deleterious gBRCAm, HER-2-negative metastatic breast cancer which failed neoadjuvant, adjuvant, or metastatic chemotherapy [29]. Τhe same year, talazoparib was granted approval by the FDA for patients with deleterious or potentially deleterious gBRCAm, HER-2-negative locally advanced or metastatic breast cancer, given the EMBRACA phase III trial outcomes [30]. The favorable effect on metastatic disease fostered the research of PARP inhibitors also in the early-stage disease. Talazoparib, specifically, is deemed the most potent PARP inhibitor candidate for neoadjuvant single-agent treatment [31]. Additional PARP inhibitors for breast cancer, such as niraparib, rucaparib, and veliparib, are still tested in the clinical setting either as monotherapy and as combinatory options [32].

PD-L1 protein - immune checkpoint inhibitors

PD-L1 is frequently expressed in one out of five patients with TNBC and has been associated with distinct features of breast cancer, such as younger age, large tumor size, high grade, and significant proliferation. PD-L1 is expressed on approximately 10% of cancer cells and up to 65% of tumor-infiltrating immune cells(IC) [33]. The immunotherapy agent atezolizumab, marketed as Tecentriq, combined with the immunotherapy regimen albumin-bound paclitaxel or nab-paclitaxel, branded as Abraxane, received approval for the treatment of unresectable locally advanced or metastatic triple-negative, PD-L1-positive breast cancer [28]. 

The employment of immune checkpoint inhibitors for the stimulation of the immune cells and the tumor growth limitation plays a key role in enhancing the treatment of TNBC. Atezolizumab, an anti-PD-L1 inhibitor, has been the first of such regimens to receive the FDA accelerated approval in March 2019. The approval was obtained due to the sustained efficacy it provided (25 months vs. 18 months) as an adjuvant to nab-paclitaxel compared to nab-paclitaxel alone in patients with metastatic TNBC and IC PD-L1 expression (PD-L1+ IC ≥ 1%) (IMpassion 130 trial) [34]. Moreover, in November 2020, based on the KEYNOTE-355 trial, the FDA granted accelerated approval to pembrolizumab (Keytruda) as an adjuvant to chemotherapy for the management of locally relapsed unresectable or metastatic TNBC with positive PD-L1 expression [35]. Finally, durvalumab (Imfinzi) combined with neoadjuvant anthracycline and taxane-based chemotherapy substantially improved survival rates in patients with early TNBC, according to GeparNUEVO phase II trial, as announced in the 2021 American Society of Clinical Oncology (ASCO) Annual Meeting. The findings of the trial showed that patients in the durvalumab arm obtained a complete response of 53.4% compared to 44.2% of patients in the placebo arm [36].

Antibody-drug conjugate targeting TROP-2 - sacituzumab govitecan

Sacituzumab govitecan is an antibody-drug conjugate comprised of an antibody against an antigen expressed in most types of breast cancer, the human trophoblast cell-surface antigen 2 (TROP-2), paired with topoisomerase I inhibitor (SN-38) via a patented hydrolyzable linker [37]. On April 7, 2021, the FDA approved the use of sacituzumab govitecan (Trodelvy) for patients with unresectable locally advanced or metastatic TNBC who have received previously two or more systemic treatments, at least one of them for metastatic cancer [38].

In the ASCENT phase III clinical trial, a total of 468 patients without brain metastases were randomized to either receive sacituzumab govitecan or chemotherapy. Results showed that the median progression-free survival was nearly six months with sacituzumab govitecan and less than two months with chemotherapy, while sacituzumab govitecan outperformed chemotherapy in the overall survival as well (12.1 months vs. 6.7 months). However, sacituzumab govitecan demonstrated a less satisfactory safety profile regarding treatment-related adverse events of grade 3 or higher than chemotherapy, with neutropenia, leukopenia, diarrhea, anemia, and febrile neutropenia being the most common [37].

## Conclusions

The development of novel regimens for the management of TNBC has demonstrated extraordinary progress over recent years. Unlike in the past, when chemotherapy was the only available option, immunotherapy and targeted inhibitors have been added to the therapeutic armamentarium as part of the treatment approach for advanced TNBC. They are currently under research for the management of early-stage disease too. The ideal sequencing of such regimens is not determined yet and may gradually present a challenge in the real-life clinical setting as further biologic agents will gain approval for TNBC. Finally, a number of additional treatments that explicitly target key pathways of TNBC biology are under development. The development of those agents may alter the TNBC therapeutic landscape in the foreseeable future. Conclusively, these novel lines of research and treatment options will lead to a customized, patient-centered therapeutic approach for both early-stage and advanced TNBC. Treatment decision-making will be guided by specific predictive biomarkers and medications that augment efficacy and improve the safety profile. As a result, the treatment paradigm for TNBC will evolve significantly in the future, improving the survival and patients’ quality of life.
